# Performance of India’s national publicly funded health insurance scheme, Pradhan Mantri Jan Arogaya Yojana (PMJAY), in improving access and financial protection for hospital care: findings from household surveys in Chhattisgarh state

**DOI:** 10.1186/s12889-020-09107-4

**Published:** 2020-06-16

**Authors:** Samir Garg, Kirtti Kumar Bebarta, Narayan Tripathi

**Affiliations:** State Health Resource Centre, Raipur, Chhattisgarh India

**Keywords:** Universal health coverage, Publicly funded health insurance, Purchasing, Financial protection, Access, PMJAY, India, LMIC health systems, Hospital care

## Abstract

**Background:**

A national Publicly Funded Health Insurance (PFHI) scheme called Pradhan Mantri Jan Arogaya Yojana (PMJAY) was launched by government of India in 2018. PMJAY seeks to cover 500 million persons with an annual cover of around 7000 USD per household. PMJAY claims to be the largest government funded health scheme globally and has attracted an international debate as a policy for Universal Health Coverage. India’s decade-long experience of the earlier national and state-specific PFHI schemes had shown poor effectiveness in financial protection. Most states in India have completed a year of implementation of PMJAY but no evaluations are available of this important scheme.

**Methods:**

The study was designed to find out the effect of enrolment under PMJAY in improving utilisation of hospital services and financial protection in Chhattisgarh which has been a leading state in implementing PFHI in terms of enrolment and claims. The study analyses three repeated cross-sections. Two of the cross-sections are from National Sample Survey (NSS) health rounds – year 2004 when there was no PFHI and 2014 when the older PFHI scheme was in operation. Primary data was collected in 2019-end to cover the first year of PMJAY implementation and it formed the third cross-section. Multivariate analysis was carried out. In addition, Propensity Score Matching and Instrumental Variable method were applied to address the selection problem in insurance.

**Results:**

Enrollment under PMJAY or other PFHI schemes did not increase utilisation of hospital-care in Chhattisgarh. Out of Pocket Expenditure (OOPE) and incidence of Catastrophic Health Expenditure did not decrease with enrollment under PMJAY or other PFHI schemes. The size of OOPE was significantly greater for utilisation in private sector, irrespective of enrollment under PMJAY.

**Conclusion:**

PMJAY provided substantially larger vertical cover than earlier PFHI schemes in India but it has not been able to improve access or financial protection so far in the state. Though PMJAY is a relatively new scheme, the persistent failure of PFHI schemes over a decade raises doubts about suitability of publicly funded purchasing from private providers in the Indian context. Further research is recommended on such policies in LMIC contexts.

## Background

The Ayushman Bharat - Pradhan Mantri Jan Arogaya Yojana (PMJAY) was launched by the central government of India in 2018 as a national Publicly Funded Health Insurance (PFHI) scheme [1–3]. PFHI schemes have been seen as important means to achieve Universal Health Coverage (UHC) in LMICs, including India [4–6]. PMJAY replaces the earlier national PFHI scheme known as the Rashtriya Swasthya Bima Yojana (RSBY) which was in operation for a decade till 2018 [1]. PMJAY claims to be the largest health insurance or assurance scheme in the world, fully financed by government [1]. PMJAY seeks to cover 500 million persons with an annual cover of half a million Indian Rupees (around 7000 USD) per household [1]. It provides a seventeen times larger vertical cover as compared to RSBY [1].

The launch of PMJAY as a key policy in Indian healthcare has been hailed as an important step towards UHC [6]. It has also attracted a vigorous international debate on its merits and suitability for Indian health system [6–13]. The experience of RSBY in India has been well documented and its effectiveness in financial protection was found to be poor [14–24]. The evidence on impact of RSBY in improving access to hospital care has been mixed [15–20]. Some states in India have implemented their own PFHI schemes with cover around 7 times bigger than RSBY [1, 25, 26]. The recent evidence has shown their poor effectiveness in improving access and financial protection though the evidence from some earlier studies has been more mixed [26–30].

Around 90% of the states in India had agreed to join hands with the central government to implement PMJAY. Most states have completed a year of implementation of PMJAY [3]. PMJAY, in first year of its implementation, issued 103 million enrollment cards, empanelled 18,236 hospitals and was utilized for over 4.6 million hospitalizations nationally [3]. However, no evaluations are available on how this important scheme has performed in meeting its key objectives.

The stated objectives of PMJAY are to reduce the financial burden on poor and vulnerable groups for access to quality health services [1]. It covers most of the hospital based secondary and tertiary care [1]. PMJAY has defined around 1370 medical packages covering surgery and treatments including medicines and diagnostics, pre and post-operative care, food and accommodation [1]. PMJAY aims to build on the base provided by RSBY, the national PFHI scheme implemented by many states during 2008 to 2018 [2]. The design of PMJAY differs from RSBY in two key features:

- Annual sum assured per family under PMJAY is around USD 7000, whereas it was USD 420 under RSBY. Providing an adequately large annual cover has been the central rationale stated by national government for moving from earlier schemes to PMJAY [1]. PMJAY covers a larger range of medical procedures as compared to RSBY.
- PMJAY covers all the individuals in households classified as poor or vulnerable under the national census. The eligibility under RSBY was relatively restricted as it did not allow more than 5 members per family to get enrolled [1, 3].

Like RSBY, patients enrolled under PMJAY are also not supposed to pay any part of the healthcare cost at any stage [1–3]. The services under PMJAY are expected to be completely free for the enrolled persons and “cash-less” at the point of care [1, 2]. The implementation arrangements of PMJAY are similar to RSBY or state based PFHI schemes [1–3]. Under PMJAY, states empanel a mix of private and public hospitals to provide a package of in-patient services at pre-defined prices. Some states engage private or public insurance firms as intermediaries. Other state governments set up their own ‘Trusts’ to as a purchaser organisation [1, 3].

Chhattisgarh state in central India has been among the leading states in implementing PMJAY in terms of population-enrolment as well as utilisation [3]. Chhattisgarh was a leading state in implementing RSBY as well, earlier [19]. The state started implementing PFHI in form of RSBY in 2009 [19]. RSBY covered the poor households and provided them an annual cover of 420 USD per family. In 2012, Chhattisgarh expanded the population coverage under PFHI by providing additional state funding for covering the non-poor households [19]. The implementation of PMJAY in Chhattisgarh state started from September 2018 [3]. While PMJAY covered the poor, the non-poor were covered through another smaller PFHI scheme known as Mukhyamantri Swasthya Bima Yojana (MSBY) [31]. PMJAY had an annual cover of around 7000 USD per family whereas MSBY cover was much smaller at 700 USD per family. Around 90% of the households and 70% of the individuals living in the state were enrolled under the two schemes with PMJAY contributing to around two-third of the individuals covered [19, 31]. There were 937 hospitals empanelled and a majority of them were for-profit private providers [32]. Private providers accounted for around 85% of the claim amount under PMJAY, as was the case under RSBY in Chhattisgarh [33]. The above feature is a continuation of pattern prevalent under earlier PFHI schemes in Chhattisgarh and most states in India [26]. Though options of getting insured under mechanisms other than PFHI are available, a very small proportion of population is enrolled under them in Chhattisgarh [19].

This study is aimed at evaluating the performance of PMJAY in improving utilisation and financial protection for hospital care in Chhattisgarh state.

### Methods

For evaluating PFHI schemes, literature recommends that using observations of more than one time is ideal, with one measurement before the insurance scheme began [34]. This study uses three repeated cross-sections. It utilized the opportunity available through the dataset of the National Sample Survey (NSS) of India in form of its two cross-sectional datasets. The 60th round of NSS provides data on hospital care and OOPE for year 2004, which was before PFHI programmes were introduced in any state of India [35, 36]. The 71st round of NSS in 2014 provides data when RSBY i.e. the scheme with 420 USD annual cover per family was in operation [37–39]. Since there was no household survey available for period after implementation of PMJAY scheme started in September, 2018, a primary survey was carried out in Chhattisgarh in October–November 2019, to collect data on hospital care that took place during first year of implementation of PMJAY. The primary survey of 2019 was carried out on NSS lines. Table 1 provides a summary of PFHI schemes at the three cross-sections.

**Table 1:** PFHI schemes and annual cover in Chhattisgarh State: three cross-sections

Year	PFHI Scheme	Annual cover per family	Eligible group
2004	None	None	None
2014	RSBY	USD 420	a) Poor Households (supported by central government funding) b) Non-Poor Households (fully funded by state government)
2019	PMJAY	USD 7000	Poor Households (supported by central government funding)
	MSBY	USD 700	Non-Poor Households (fully funded by state government)

NSS is conducted by the National Sample Survey Office of the central Ministry of Statistics. This institution was established in1950 by Government of India. NSS conducts annual surveys but the topics vary from round to round. The main areas on which surveys have been conducted under NSS include: Employment, Consumer Expenditure, Informal and Formal Enterprises, Investments and Debt, Education and Healthcare. Researchers have recommended the use of NSS for public health research [40]. The current study uses the data from Survey Rounds on Morbidity and Healthcare, which were carried out in 2004 and 2014 (Round 60th and 71st) respectively. The above datasets have been widely used in peer-reviewed research on healthcare in India, including on government health insurance schemes [18–20, 26]. The 60th and 71st rounds are similar in design, content and coverage, and thereby provide comparable results [41]. Their sample is population based and nationally representative [41]. Researchers have observed that the above rounds provide comprehensive information on morbidity patterns, health care, type of providers (public or private), out of pocket expenditure for hospitalization [41, 42]. NSS has reported adequate respondent-cooperation rates in its 60th and 71st rounds. The information on responses for Chhattisgarh found from the NSS data-sets and the 2019 survey in Chhattisgarh is presented in Table 2:

**Table 2:** Responses and cooperation rates in household surveys

Response	NSS 60th Round - 2004 Chhattisgarh Data	NSS 71st Round - 2014 Chhattisgarh Data	Chhattisgarh Survey-2019
	Informants (%) *N* = 1470	Informants (%) *N* = 1205	Informants (%) *N* = 2612
Co-operative and capable	69.7	78.7	85.4
Co-operative but not capable	28.7	19.1	11.7
Busy	1.3	2.1	2.5
Reluctant	0.1	0.02	0.3
Others	0.09	0	0

There were no rounds of surveys by NSS on morbidity and healthcare between 2004 and 2014 [41]. A new round on health was conducted in 2017 (75th round) [42]. However, it could not be included in the current study. The dataset of the 75th round became available only by end of 2019 when the current study was in its advanced stages.

The primary survey in 2019 followed a two-stage stratified sampling similar to NSS. A detailed note on the sample design is available in NSS documents [35–39]. The sampling weights were taken into account in the analyses as applicable. In Chhattisgarh, NSS survey covered 6375 individuals in 2004 and 7651 individuals in 2014. The primary survey carried out in Chhattisgarh in 2019 covered 15,361 individuals. Since the main objective of the study was to measure the change in financial protection for hospital care, an adequate number of hospitalization episodes was needed in the sample. The two-stage design was taken into account in calculating the sample-size. For a detectable difference of 5% at 95% confidence and a design effect of 1.5, a requirement of around 570 hospitalization episodes was calculated. The actual number of hospitalization episodes covered in the NSS sample was 556 and 817 for year 2004 and 2014 respectively. The 2019 survey was able to cover 924 hospitalisations. The size of sample available was therefore adequate to detect difference of 5%.

Financial Protection was measured in terms of Catastrophic Health Expenditure (CHE) as proposed by Wagstaff and Doorslaer [43]. Out of Pocket Expenditure (OOPE) was calculated for each episode by adding medical expenses and expenses on transportation and deducting any cash-reimbursements received by the patient. OOPE amounts for 2004 and 2019 were adjusted at 2014 prices for valid comparison, as done by recent studies [26, 44]. For the above adjustment, price deflators for rural (agricultural labour) and urban areas (industrial workers) were used [44, 45]. The survey collected data on usual monthly consumption expenditure and it was multiplied by twelve to calculate the Usual Annual Consumption Expenditure. Recent studies analyzing the NSS datasets have used the same procedure for calculating Annual Household Consumption Expenditure [16, 26]. Thresholds of 10, 25 and 40% of concerned household’s Annual Consumption Expenditure were taken for CHE and named CHE10, CHE25 and CHE40 respectively. Improvement in access to hospital care was assessed in terms of change in utilisation of hospital care.

The survey data was analysed using STATA V.14. Multivariate analysis was carried out to find effect of PMJAY on utilisation, OOPE and CHE. It compared those enrolled under PMJAY with the rest. This analysis was repeated to find out effect of enrollment under any PFHI scheme – PMJAY, MSBY or RSBY, by comparing with the individuals not-enrolled in PFHI. The list of variables in the study is given in Additional file S1. The variables mentioned in S1 were controlled in the multi-variate models.

Ordinary Least Squares (OLS) was applied for continuous outcome variables (OOPE, Log of OOPE). Probit model was used for binary outcome variables (CHE). For robustness, this was compared with the Average Treatment Effect on the Treated (ATET) under Propensity Score Matching (PSM) modeling. PSM and other forms of ‘matching’ have been used for evaluating PFHI, including in India [16, 20].

In addition to the above, multivariate analysis for OOPE and CHE was repeated using the Instrumental Variable (IV) approach. This additional analysis was carried out to address the potential selection problem or endogeneity, known to be a common issue while finding out the effect of insurance on economic outcomes like OOPE [34, 46, 47]. Instrumental Variable (IV) method has been recommended as a robust solution to the potential problem of endogeneity [34, 46–49]. IV method has been applied in evaluations of impact of PFHI schemes on OOPE and CHE in India, Mexico, China and Ghana [26, 47, 50, 51].

Two-stage least squares (2sls) was applied as IV model for OOPE and Two-step IV Probit for CHE [26, 49, 52]. Wu-Hausman test for 2sls and Wald test of exogeneity for IV Probit were conducted to test for endogeneity. ‘Marital-status’ was used as an instrumental variable because it satisfied both the criteria for a suitable IV – it was associated with scheme-enrolment and was not expected to have a direct impact on outcome i.e. OOPE. Over-identification restriction tests were applied to check the validity of IV model chosen [52, 53]. The results of the above tests have been reported along with the regression results. A brief note on endogeneity and IV approach is given in Additional file S2.

Significance was taken at 95% (*p* < 0.05).

### Findings

The sample profile is given in Additional file S3.

### Enrollment under PFHI

Enrollment rate under PFHI has increased in Chhattisgarh over the years. Table 3 provides the scheme-wise descriptive findings on enrollment and hospital-utilisation.

**Table 3:** Descriptive findings on enrolment and hospitalization under different PFHI schemes in Chhattisgarh

**a. Proportion of individuals (%) enrolled under different PFHI schemes in Chhattisgarh with 95%CI in ()**			
**PFHI scheme**	**Year 2004**	**Year 2014**	**Year 2019**
***N***	6375	7651	15,361
**RSBY**		38.8 (37.5–40.0)	
**PMJAY**			45.8 (45.0–46.1)
**MSBY**			22.0 (21.3–22.6)
**Not Enrolled in PFHI**	100 (100–100)	61.3 (60–62.5)	32.1 (31.4–32.9)
**b. Proportion (%) of individuals in Chhattisgarh who utilized hospital care with 95% Confidence Intervals in ()**			
	**2004**	**2014**	**2019**
All	1.4 (1.1–1.6)	3.0 (2.6–3.4)	5.9 (5.6–6.3)
PFHI-enrolled		3.3 (2.6–4.0)	6.0 (5.6–6.5)
Not Enrolled in PFHI	1.4 (1.1–1.6)	2.9 (2.3–3.4)	5.7 (5.1–6.4)
**c. Proportion of Hospitalisation episodes in private hospitals (%) with 95% CI in ()**			
**Insurance Status**	**Year 2004**	**Year 2014**	**Year 2019**
**N**	556	817	924
**All**	47.5 (43.4 to 51.7)	44.8 (41.5 to 48.2)	39.9 (36.7 to 43.1)
**PFHI-enrolled**		32.8 (28.1 to 37.5)	45.1 (41.1 to 49)
**Not Enrolled in PFHI**	47.5 (43.4 to 51.7)	53.6 (49.1 to 58)	28.6 (23.3 to 33.9)

### Utilization of hospital care

The hospitalization rate has increased over the years in Chhattisgarh, for the PFHI-enrolled as well as the individuals not-enrolled in PFHI (Table 3).

The naïve Probit model as well as PSM showed no significant association between hospitalization and PMJAY-enrollment in Chhattisgarh (Additional file S4). However, utilisation was negatively associated with PFHI-enrolment according to the naïve model (Additional file S4). When PSM was applied, it showed that the effect of PFHI-enrollment on utilisation was almost negligible. Table 4 provides a summary of findings on effect of enrollment on utilisation.

**Table 4:** Effect of enrolment under PMJAY and PFHI on Utilisation of Hospital Care – Results of Naïve (Probit) model and PSM model

Model	PMJAY		PFHI	
	Coeff.	*P*	Coeff.	*P*
Probit	−0.02	0.54	−0.17*	**< 0.01**
PSM	0.003	0.33	−0.01*	**< 0.01**

*p < 0.05

### Choice of provider

Among hospitalizations in each survey-round, more than 50% had utilized public sector. In 2019, public sector accounted for over 60% of the utilisation. The share of private sector in the hospitalizations of the PFHI-enrolled increased from 2014 to 2019 while it declined for the non-enrolled in PFHI during that period (Table 3).

### OOPE and financial protection

The mean OOPE for utilizing hospital-care in private sector was many times larger than in public sector. This was true for the PFHI-enrolled and also for the non-enrolled in PFHI (Table 5). The mean OOPE was similar for the PMJAY-enrolled and for the non-enrolled in PMJAY.

**Table 5:** Descriptive findings on OOPE and CHE25 under different PFHI schemes

**a. Mean OOPE for Hospitalisation Episodes (in INR) with 95% CI in ()**				
**PFHI scheme**	**Type of hospital**	**Year 2004**	**Year 2014**	**Year 2019**
	**n**	556	817	924
All	Public	8603 (6818–10,388)	3491 (2844–4137)	3101 (2281–3922)
	Private	15,280 (12195–18,365)	22,929 (18481–27,377)	26,108 (18622–33,595)
RSBY	Public		2633 (1669–3598)	
	Private		26,326 (17734–34,918)	
PMJAY	Public			3078 (1928–4228)
	Private			19,375 (11305–27,447)
MSBY	Public			3506 (920–6092)
	Private			41,154 (20689–61,619)
Not enrolled	Public	2912 (2213–3749)	1800 (1537–2000)	2974 (1675–4272)
	Private	7922 (6647–9407)	13,650 (10500–16,778)	20,261 (11689–28,843)
**b. Median OOPE for Hospitalisation Episodes (in INR) with 95% CI in ()**				
**PFHI scheme**	**Type of Hospital**	**Year 2004**	**Year 2014**	**Year 2019**
	**n**	556	817	924
All	Public	2912 (2213–3749)	1100 (903–1350)	378 (378–606)
	Private	7922 (6647–9407)	12,450 (10500–15,222)	7575 (7299–10,253)
RSBY	Public		570 (400–800)	
	Private		10,650 (9510–15,093)	
PMJAY	Public			530 (379–758)
	Private			7299 (3788–9032)
MSBY	Public			303 (151–496)
	Private			13,447 (7299–18,138)
Not enrolled	Public	2912 (2213–3749)	1800 (1537–2000)	417 (298–703)
	Private	7922 (6647–9407)	13,650 (10500–16,778)	8759 (7575–11,990)
**c. Proportion of incurred CHE25 for Hospitalisation Episode (%) with 95% CI in ()**				
**PFHI scheme**	**Type of Hospital**	**Year 2004**	**Year 2014**	**Year 2019**
	**n**	556	817	924
All	Public	14.9 (10.6–19.3)	4.4 (2.5–6.3)	7.2 (5.0–9.4)
	Private	27.6 (22.6–32.9)	32.1 (27.6–36.6)	39.4 (34.4–44.5)
RSBY	Public		4.8 (2.0–7.7)	
	Private		34.4 (26.9–41.8)	
PMJAY	Public			7.6 (4.5–11.0)
	Private			43.6 (36.3–51.4)
MSBY	Public			3.8 (0.5–8.1)
	Private			32.4 (23.4–41.4)
Not enrolled	Public	14.9 (10.6–19.3)	4.0 (1.4–6.6)	7.9 (4.2–11.7)
	Private	27.6 (22.6–32.9)	30.7 (25.0–36.4)	39.5 (28.6–50.4)

Median OOPE in the private sector was many times greater than in public sector (Table 5). According to descriptive findings, median OOPE in private hospitals for the PMJAY-enrolled was 17% lower than for the non-enrolled in PMJAY.

### CHE25 incidence

CHE25 incidence was many times greater for utilisation in private sector as compared to public sector. CHE25 incidence was similar for the PMJAY-enrolled and for the non-enrolled in PMJAY (Table 5).

### Determinants of size of OOPE and CHE

Table 6 provides a summary of findings regarding the effect of PMJAY and all PFHI schemes respectively on OOPE and CHE. The naïve OLS model showed no association between the size of OOPE and enrollment under PMJAY or any of the PFHI schemes. The above finding did not change under PSM and IV models.

**Table 6:** Effect of enrolment under PMJAY and PFHI on OOPE and CHE for Hospital Care – Results of Naïve (Probit) model, PSM and IV Models

Variable	Scheme	OLS model		Probit model		PSM model (ATET)		IV Model	
		Coeff.	*P*	Coeff.	*P*	Coeff.	*p*	Coeff.	*P*
OOPE	PMJAY	− 4287	0.09			− 4614	0.20	48,734	0.59
	PFHI	−87	0.97			− 1066	0.73	17,315	0.72
Log of OOPE	PMJAY	−0.45*	**< 0.01**			−0.37*	**< 0.01**	−0.48	0.86
	PFHI	−0.34*	**< 0.01**			− 0.50*	**< 0.01**	1.01	0.53
CHE10	PMJAY			0.08	0.35	0.02	0.52	−4.39	0.28
	PFHI			−0.07	0.29	0.003	0.90	−2.23	0.23
CHE25	PMJAY			0.22*	**0.01**	0.05	0.08	−2.03	0.54
	PFHI			0.04	0.56	0.02	0.33	−1.28	0.48
CHE40	PMJAY			0.26*	**0.01**	0.04	0.14	−0.67	0.85
	PFHI			0.05	0.55	0.01	0.36	−0.68	0.74

*p < 0.05

The naïve as well as the PSM model showed a significant but small reduction in Log of OOPE associated with PMJAY enrolment. The IV model however showed no association between Log of OOPE and PMJAY enrolment.

PMJAY enrolment was associated with increase in CHE25 and CHE 40 according to the naïve Probit model. However, the above finding did not hold under PSM and the IV models. CHE10 was not associated with PMJAY or PFHI enrollment under any of the models.

Under all the models, significantly greater OOPE and CHE were likely for utilisation in private sector as compared to public sector. NCDs or Injuries compared to Communicable diseases and hospitalisations longer than 3 days were also associated with greater OOPE or CHE (Additional files S5 to S14).

The OLS models for OOPE and Log of OOPE is given in Additional files S5 and S6 respectively. The IV models for OOPE and Log of OOPE is given in Additional files S7 and S8 respectively. The naïve Probit models for CHE10, CHE25 and CHE40 are given in Additional files S9, S10 and S11 respectively. The IV Models for CHE10, CHE25 and CHE40 are given in Additional files S12, S13 and S14 respectively.

For robustness, the above analysis were repeated with the single cross-section data from 2019 primary survey and the pattern of the results remained similar for effect of PMJAY on OOPE or CHE.

## Discussion

The utilisation of hospital care did not increase with enrollment under PMJAY or other PFHI schemes in Chhattisgarh. Some earlier studies have concluded that utilisation increased due to PFHI in India [17, 19, 20]. The mixed findings could be due to differences in the methods applied and time-periods of different studies. The more recent studies that have applied PSM or IV to address selection issues, have reported no increase in utlisation with PFHI enrolment [16, 26].

The current study found that coverage under PMJAY or other PFHI schemes in Chhattisgarh did not reduce OOPE or CHE. The inability of PFHI in ensuring financial protection for hospital-care is consistent with many other studies of RSBY and other state-level PFHI schemes in India [15–26]. Some studies had suggested that the vertical cover of INR 30,000 (USD 420) annual sum assured per family might be insufficient, thereby causing possibility of CHE under RSBY [15, 19]. PMJAY design of a seventeen times larger sum assured was expected to reduce OOPE, but the current study found that it failed to do so. A study of Southern states in India had shown that a large cover may not ensure financial protection [26].

Why did OOPE and CHE remain high under PMJAY? The benefit stipulated in PMJAY and other PFHIs was of free cashless service covering pre and post operative care, diagnostics, drugs and transportation. The contracts forbade the hospitals from charging any copayments. Yet, the mechanism of contracting could not prevent private hospitals from taking extra money from patients. The size of OOPE and incidence of CHE in the current study was several times higher for private-sector hospitalizations irrespective of enrollment under PFHI, as found in earlier studies in India [16, 19, 26]. The likelihood of insurance benefit being appropriated by powerful providers has been a long-standing problem in LMIC contexts [54]. The possibility of ‘provider capture’ has been pointed out [12]. Researchers have also highlighted the role of unnecessary or costly medical procedures being used by providers under such schemes [8, 12].

Some studies have found ‘double-billing’ by hospitals as a cause of OOPE under PFHI in India [24, 25]. ‘Double billing’ in the context of PFHI has been referred to the situation when hospitals, while claiming the amount for a service from insurance side, also charged illegal copayments from patients for the same service or asked them to buy drugs, diagnostics and consumables from outside [24–26]. Tendencies to charge extra from the patients, despite PFHI cover have been reported from several states of India [24–26]. Studies have recommended that stronger supervision by state authorities, better mechanisms for addressing grievances and a 24-h helpline should be implemented to address the problem of ‘double-billing’ [15, 16, 24]. PMJAY included measures for fraud control and a 24-h helpline but they seem to be ineffective as far as protecting patients from extra charging is concerned. A recent qualitative study of experiences of patients of utilizing private sector care under PFHI in Chhattisgarh has provided fresh insights about the regulatory failure as well as the normative and cultural contexts contributing to over-charging by private hospitals [55]. We recommend further qualitative research, including on the regulation and purchasing side, to understand the gaps leading to poor financial outcomes under PFHI.

The current study suggests that the main challenges faced by earlier PFHI schemes in India continue to plague PMJAY. This scheme does have a bigger vertical cover and offers a larger range of treatment packages compared to other schemes. But, little else seems to be different in terms of design features or implementation arrangements. There seems to be a failure in recognizing the reasons for failures of previous schemes and devising measures to address them in PMJAY.

The study examined the first year of implementation of PMJAY and the scheme would require serious changes if it has to meet its objectives in coming years. PMJAY is a relatively new scheme but India now has more than a decade of experience in implementing PFHI based purchasing. Its persistent lack of success in financial protection indicates the limitations of PFHI strategy in Indian context. Some of the states in India, e.g. Odisha and Delhi, have chosen to stay out of PMJAY and have implemented differently designed health schemes of their own. It might be useful to study such models and compare their outcomes with PMJAY in other states.

The unacceptably high OOPE under PMJAY may be related to provider behavior and continuing poor regulation. There is a growing recognition that governance and control needs to be strong for purchasing to be successful in LMICs [56, 57]. The relative size of OOPE in private and public sector hospitals, suggests that the share of the public sector could be increased in provisioning to bring down overall OOPE [26]. Further research is recommended on experiences of publicly funded schemes in LMICs that rely on contracting for-profit providers.

### Limitations

The NSS dataset does not distinguish between older insurance schemes of Central Government Health Services (CGHS) and Employee State Insurance (ESI) for the formally employed and the current wave of PFHIs that were the focus of this study. Other studies have reported that CGHS and ESIS form a very small proportion of PFHI enrollment and do not affect the results materially [16, 19]. The health-condition of patients can be a factor in healthcare utilisation and expenditure but data was not available on this aspect in the surveys used.

## Conclusions

The study provides one of the first evaluations of PMJAY, the latest national health insurance programme in India. Based on above analysis, we conclude there was either an insignificant or at best a minor reduction in OOPE with PMJAY-enrollment, but none in CHE.

The evaluation of its first year of implementation suggests that gaps and failures of earlier PFHI schemes have persisted under PMJAY. Just increasing annual sum assured and addition of treatment packages has not given the desired results in improving access or financial protection. Major changes may be necessary in how provisioning is organized for achieving progress towards goals of UHC. Further evaluations of PMJAY and of alternative schemes in other states are recommended along with qualitative studies. PFHI based purchasing seems to have limitations as a policy option in Indian health system context. Studies of national schemes involving publicly funded purchasing are recommended for other LMICs.

## Supplementary information

**Additional file 1. Study Variables** - This table lists the variables used in the study.

**Additional file 2. A note on Instrumental Variable (IV) method** - This provides a brief note on the problem of endogeneity and the IV as a method of addressing it.

**Additional file 3. Sample Profile** - This table provides the demographic and socio-economic profile of surveyed individuals.

**Additional file 4. Probit Model for Utilisation** – This file has two tables. Table S4.1 provides Probit Model for Utlisation with PMJAY-enrolment as one of the controlled variables. Table S4.2 provides Probit Model for Utlisation with PFHI-enrolment as one of the controlled variables.

**Additional file 5. OLS Model for OOPE** – This file has two tables. Table S5.1 provides OLS Model for OOPE with PMJAY-enrolment as one of the variables. Table S5.2 provides OLS Model for OOPE with PFHI-enrolment as one of the variables.

**Additional file 6. OLS Model for log of OOPE** – This file has two tables. Table S6.1 provides OLS Model for log of OOPE with PMJAY-enrolment as one of the variables. Table S6.2 provides OLS Model for log of OOPE with PFHI-enrolment as one of the variables.

**Additional file 7. IV Model (2sls) for OOPE** – This file has two tables. Table S7.1 provides IV Model (2sls) Model for OOPE with PMJAY-enrolment as a variable. Table S7.2 provides IV Model (2sls) Model for OOPE with PFHI-enrolment as a variable.

**Additional file 8. IV Model (2sls) for log of OOPE** – This file has two tables. Table S8.1 provides IV Model (2sls) Model for log of OOPE with PMJAY-enrolment as a variable. Table S8.2 provides IV Model (2sls) Model for log of OOPE with PFHI-enrolment as a variable.

**Additional file 9. Probit Model for CHE10** – This file has two tables. Table S9.1 provides Probit Model for CHE10 with PMJAY-enrolment as a variable. Table S9.2 provides Probit Model for CHE10 with PFHI-enrolment as a variable.

**Additional file 10. Probit Model for CHE25** – This file has two tables. Table S10.1 provides Probit Model for CHE25 with PMJAY-enrolment as a variable. Table S10.2 provides Probit Model for CHE25 with PFHI-enrolment as a variable.

**Additional file 11. Probit Model for CHE40** – This file has two tables. Table S11.1 provides Probit Model for CHE40 with PMJAY-enrolment as a variable. Table S11.2 provides Probit Model for CHE40 with PFHI-enrolment as a variable.

**Additional file 12. IV Model (IVProbit) for CHE10** – This file has two tables. Table S12.1 provides IV Model for CHE10 with PMJAY-enrolment as a variable. Table S12.2 provides IV Model for CHE10 with PFHI-enrolment as a variable.

**Additional file 13. IV Model (IVProbit) for CHE25** – This file has two tables. Table S13.1 provides IV Model for CHE25 with PMJAY-enrolment as a variable. Table S13.2 provides IV Model for CHE25 with PFHI-enrolment as a variable.

**Additional file 14. IV Model (IVProbit) for CHE40**– This file has two tables. Table S14.1 provides IV Model for CHE40 with PMJAY-enrolment as a variable. Table S14.2 provides IV Model for CHE40 with PFHI-enrolment as a variable.

## Data Availability

The secondary dataset used are available at [microdata.gov.in/nada43/index.php/catalog/105] and [microdata.gov.in/nada43/index.php/catalog/135] for 2004 and 2014 rounds of NSS respectively. The combined anonymised dataset, including the 2019 survey is available in public domain at [shsrc.in/cg-health-at-a-glance/study data].

## Abbreviations

CHE: Catastrophic health expenditure
CHE10: Catastrophic Health Expenditure computed using the threshold of 10% of usual annual consumption expenditure
CHE25: Catastrophic Health Expenditure computed using the threshold of 25% of usual annual consumption expenditure
CHE40: Catastrophic Health Expenditure computed using the threshold of 40% of usual annual consumption expenditure
CI: Confidence Interval
INR: Indian Rupee
IV: Instrumental Variable
LMIC: Low and Medium Income Countries
NSS: National sample survey
OLS: Ordinary Least Squares
OOPE: Out-of-pocket expenditure
PFHI: Public Funded Health Insurance
PMJAY: Pradhan Mantri Jan Arogaya Yojana
RSBY: Rashtriya Swasthya Bima Yojana
UHC: Universal Health Coverage
USD: US Dollar
2sls: Two Stage Least Squares
