# Supplementary material for: Performance of India’s national publicly funded health insurance scheme, Pradhan Mantri Jan Arogaya Yojana (PMJAY), in improving access and financial protection for hospital care: findings from household surveys in Chhattisgarh state
Source: BMC Public Health. 2020 Jun 16;20:949. doi: 10.1186/s12889-020-09107-4 (PMC7298746; doi:10.1186/s12889-020-09107-4)
Supplement: Supplementary file 2 — Additional file 2. A note on Instrumental Variable (IV) method - This provides a brief note on the problem of endogeneity and the IV as a method of addressing it. [file 12889_2020_9107_MOESM2_ESM.docx]

**Additional File S2**

**A Note on Endogeneity and Instrumental Variable (IV) Method:**

(Note: Citation numbers given here are from the Reference List given in the Main Manuscript)

Endogeneity means that an explanatory variable correlates with the disturbance term of the regression equation. Not accounting for it can result in biased parameter estimates [47]. Endogeneity can be caused by circumstances including omitted variables, simultaneity and measurement errors [53]. Literature suggests that insurance enrolment variable can induce the problem of endogeneity while assessing its effect on economic outcomes like OOPE [47-51, 53]. When selection into the scheme is non-random, the insurance-enrollment and the error term in our estimating equation are likely to be correlated with one another. This can bias the coefficient on insurance in the out-of-pocket expenditure equation [47, 53] The Instrumental Variable (IV) method has been recommended as a robust solution to potential problem of endogeneity [34, 46-49]. IV method has been considered more suitable than Difference in Difference (DID) and Propensity Score Matching for addressing endogeneity bias due to unobserved variables [46, 48]. IV method has been applied in evaluations of impact of PFHI schemes on OOPE and CHE in India, China, Mexico and Ghana [26, 47, 50, 51].

A suitable ‘Instrumental Variable’ should satisfy the ‘relevance’ criterion i.e. it should correlate with the explanatory variable, i.e. PFHI-enrollment in this case [46, 53]. A suitable Instrumental variable should not have a direct impact on the outcome variable [53]. This restriction, also called ‘over-identifying restriction’, is tested by including each subset of instruments in the last stage regressions to see whether these instruments can be justifiably excluded from these regressions. According to literature, that test allows us to evaluate the validity of the model [34, 46, 47, 52, 53]. Wu-Hausman test for 2sls (using command “estat endog”) and Wald test of exogeneity for IV Probit were conducted to test for endogeneity. Over-identification restriction tests (command “weakiv” for two step IV Probit and “estat overid” for 2sls ) were applied to check the suitability of Instrumental Variable model chosen [52]. The results of the above tests have been reported along with the regression results. Significance was taken at 95% (p<0.05).

For IV analysis, we applied Two-step least square (2sls) for OOPE and Two-step IV Probit model for Utilisation and CHE [26, 49, 52]. Some studies on impact of insurance using IV method have applied 2sls for OOPE and Control Function tests like two-stage residual inclusion for CHE [38, 39]. For robustness, we repeated the IV regressions for CHE using 2sls and also Two Step Control Function test available in STATA for endogenous covariates. Other studies have also used comparisons with IV Probit for robustness [49]. Results of naïve and Propensity Score Matching (PSM) models were compared with results of IV models.
